# p53-armed oncolytic adenovirus induces apoptosis in pancreatic cancer-associated stellate cells via macropinocytosis

**DOI:** 10.1038/s41417-025-00989-3

**Published:** 2025-11-28

**Authors:** Takeyoshi Nishiyama, Hiroshi Tazawa, Yasuo Nagai, Ryohei Shoji, Yoshinori Kajiwara, Naoyuki Hashimoto, Yosuke Takahashi, Satoru Kikuchi, Shinji Kuroda, Toshiaki Ohara, Kazuhiro Noma, Ryuichi Yoshida, Yuzo Umeda, Hiroyoshi Y. Tanaka, Mitsunobu R. Kano, Atsushi Masamune, Yasuo Urata, Shunsuke Kagawa, Toshiyoshi Fujiwara

**Affiliations:** 1 Department of Gastroenterological Surgery, Okayama University Graduate School of Medicine, Dentistry and Pharmaceutical Sciences, Okayama, Japan; 2 Center for Innovative Clinical Medicine, Okayama University Hospital, Okayama, Japan; 3 Department of Pathology and Experimental Medicine, Okayama University Graduate School of Medicine, Dentistry and Pharmaceutical Sciences, Okayama, Japan; 4 Department of HBP and Breast Surgery, Ehime University Graduate School of Medicine, Ehime, Japan; 5 Department of Pharmaceutical Biomedicine, Okayama University Graduate School of Interdisciplinary Science and Engineering in Health Systems, Okayama, Japan; 6 Division of Gastroenterology, Tohoku University Graduate School of Medicine, Miyagi, Japan; 7 Oncolys BioPharma, Inc., Tokyo, Japan

**Keywords:** Cancer microenvironment, Pancreatic cancer, Targeted therapies

## Abstract

Pancreatic ductal adenocarcinoma (PDAC)-associated pancreatic stellate cells (PSCs) promote PDAC tumor progression. Notably, PDAC tumors display enhanced macropinocytosis, resulting in enhanced uptake of extracellular particles, including nutrients and viruses. We previously demonstrated the therapeutic potential of telomerase-specific oncolytic adenoviruses OBP-301 and p53-armed OBP-702 against human PDAC cells. However, it remains unclear whether macropinocytosis promotes the virus sensitivity of PDAC-associated PSCs. Here, we show that PSCs activated by human PDAC cells (Panc-1 and BxPC-3) exhibit enhanced sensitivity to wild-type and oncolytic adenoviruses via enhanced macropinocytosis. The virus sensitivity of PSCs was analyzed for the infectivity, replication, and cytopathic activity of wild-type and oncolytic adenoviruses. PDAC-associated PSCs were more sensitive to wild-type and oncolytic adenoviruses than were control PSCs; this sensitivity was mediated by activation of macropinocytosis. In three-dimensional (3D) culture models, p53-armed OBP-702 decreased the viability of PDAC-associated PSCs more strongly than did non-armed OBP-301, reflecting induction of p53-mediated apoptosis. Co-inoculation of PSCs enhanced the growth of PDAC tumors, an effect that was attenuated by OBP-702-mediated p53 activation in the tumor stroma. Our results suggest that p53-armed oncolytic adenovirus OBP-702 eliminates PDAC-associated PSCs via enhancement of macropinocytosis-mediated virus entry and induction of p53-mediated apoptosis.

## Introduction

Pancreatic ductal adenocarcinoma (PDAC) is a high-mortality disease. Despite recent advances in surgery, multi-agent chemotherapy, radiotherapy, and molecular targeting therapy, the relative 5-year survival rate for PDAC still is less than 10% [1]. A tumor microenvironment (TME) incorporating a desmoplastic stroma has been shown to be associated with tumor progression and therapy resistance in PDAC [2]. Cancer-associated fibroblasts (CAFs) are the most abundant components of the stromal tissues of PDAC [3]. CAFs are derived primarily from pancreatic stellate cells (PSCs) via activation by PDAC cells [4]. PDAC-associated PSCs play an important role in promoting chemoresistance, immune evasion, and tumor progression in PDAC tumors [4]. PDAC cells activate PSCs by secreting a variety of stimulatory factors, including cytokines, growth factors, and metabolites [5]. Therefore, the crosstalk between PDAC cells and PDAC-associated PSCs is a potential therapeutic target for treating PDAC tumors.

Macropinocytosis is an endocytic process that plays a crucial role in the uptake of extracellular particles and nutrients, thereby promoting metabolic adaptation [6]. The function of macropinocytosis under physiological and pathological conditions depends on cell type [7]. PDAC tumors are characterized as hypovascular tumors with dense stromata, leading to hypoxia-mediated metabolic stress [8]. In response, PDAC tumors induce macropinocytosis to increase the accumulation of essential amino acids [9]. PDAC cells harboring mutations in the *KRAS* gene activate macropinocytosis to support nutrient uptake by upregulating the KRAS signaling pathway [10, 11]. Recently, macropinocytosis has been shown to be activated in PDAC-activated CAFs, thereby promoting the survival of PDAC cells by increasing the supply of amino acids [12]. Therefore, PDAC cells and CAFs may cooperatively activate macropinocytosis for adaptation to the metabolic stress associated with PDAC.

Oncolytic adenovirus therapy has been emerging as a novel antitumor modality to induce tumor-specific cell death [13]. We previously developed a telomerase-specific, replication-competent oncolytic adenovirus, OBP-301, in which the *hTERT* (*human telomerase reverse transcriptase*) gene promoter drives the expression of the viral *E1A* and *E1B* genes [14, 15]. OBP-301 induces lytic cell death in both the epithelial and mesenchymal types of tumor cells [14–16]. To further enhance the therapeutic potential of OBP-301, we generated a tumor suppressor p53-armed version of the virus, designated OBP-702 [17]. OBP-702 exhibits a more profound antitumor effect than OBP-301 in both the epithelial and mesenchymal types of tumor cells, including PDAC cells, via induction of apoptosis and autophagy [17–19].

CAFs have been shown to exhibit enhanced macropinocytosis [12]. Macropinocytosis is induced by adenovirus serotype 2/5 (Ad2/5) to assist viral endocytosis in hematopoietic and epithelial cells and fibroblasts [20, 21]. However, it remains unclear whether the activation of macropinocytosis promotes the sensitivity to oncolytic adenoviruses of PDAC-associated PSCs. We hypothesized that the sensitivity to oncolytic adenoviruses exhibited by PDAC-associated PSCs reflects macropinocytosis-mediated potentiation of virus entry.

In the present study, we investigated the therapeutic potential of the tumor-specific oncolytic adenoviruses OBP-301 and p53-armed OBP-702 against PDAC-associated PSCs. PSCs were activated by incubating human PSCs with the conditioned medium (CM) derived from the human PDAC cell lines Panc-1 and BxPC-3. The resulting PDAC-associated PSCs were analyzed for their virus sensitivity, as assessed by measuring the infectivity, replication, and therapeutic effects of wild-type and oncolytic adenoviruses. The role of macropinocytosis in the virus sensitivity of PDAC-associated PSCs was assessed in the presence of an inhibitor of macropinocytosis. Additionally, the effects of oncolytic adenoviruses were analyzed using three-dimensional (3D) culture models and in vivo xenograft tumor models containing PDAC cells and PSCs.

## Materials and methods

### Cell lines

The human PDAC cell lines Panc-1 and BxPC-3, human non-small cell lung cancer cell line H1299, and human breast cancer cell line MDA-MB-231 were obtained from the American Type Culture Collection (Manassas, VA, USA). The human non-small cell lung cancer cell line A549 and human gastric cancer cell line NUGC4 were obtained from the Japanese Collection Research Bioresource (Osaka, Japan). The human normal fibroblast cell line WI38 was obtained from the Human Science Research Resources Bank (Osaka, Japan). The primary human PSC cell lines hPSC-5 and hPSC-14 were obtained from the RIKEN BioResource Research Center (Tsukuba, Ibaraki, Japan). Following recovery from frozen stocks, cells were cultured for no more than 5 months before repeating the recovery from frozen stocks. Authentication of cell identity was not performed by the authors. Panc-1 cells were maintained in Dulbecco’s Modified Eagle’s Medium (DMEM) supplemented with 10% fetal bovine serum (FBS). BxPC-3, H1299, and NUGC4 cells were maintained in RPMI 1640 medium supplemented with 10% FBS. MDA-MB-231 cells were maintained in Leibovitz’s L-15 medium supplemented with nonessential amino acids and 10% FBS. A549 cells were maintained in Eagle’s Minimal Essential Medium (EMEM) supplemented with 10% FBS. WI38 cells were maintained in EMEM supplemented with 10% FBS. hPSC-5 and hPSC-14 were maintained in a 1:1 mixture of DMEM/Ham’s F-12 supplemented with 10% FBS. All media were supplemented with 100 U/mL penicillin and 100 μg/mL streptomycin. Cultures were routinely propagated at 37 °C in a humidified 5% CO_2_ atmosphere.

### Recombinant adenoviruses

The recombinant telomerase-specific, replication-competent adenovirus OBP-301 (suratadenoturev), in which the promoter element of the *hTERT* gene drives the expression of the viral *E1A* and *E1B* genes, was constructed and characterized as described previously [14, 15]. For monitoring the infectivity of OBP-301, we generated OBP-401, in which a *GFP* (*green fluorescent protein*) expression cassette was inserted into the E3-encoding region of OBP-301 [22]. For OBP-301-mediated induction of expression of the exogenous *p53* gene, we generated OBP-702, in which a human wild-type *p53* expression cassette (under the control of the viral *Egr-1* promoter) was inserted into the E3-encoding region of OBP-301 [17]. OBP-301 is a derivative of the recombinant Ad5; in some experiments, Ad5 itself was employed as a control, serving as another type of replication-competent adenovirus. Replication-deficient adenoviral vectors encoding p53 (Ad-p53) or GFP (Ad-GFP) were used to induce p53 and GFP expression in infected cells. Recombinant viruses were purified by ultracentrifugation using cesium chloride step gradients; viral titers of the resulting stocks were determined via a plaque-forming assay using 293 cells, and these stocks were stored at −80 °C.

### Collection of conditioned medium (CM)

Human normal and cancer cells were seeded in 6-well plates at a density of 5 × 10^5^ cells/well; the plates then were incubated for 24 h. After washing with phosphate-buffered saline (PBS), serum-free medium (SFM) was added to the wells, and the plates were incubated for 48 h. Following centrifugation, the resulting supernatant (the spent culture medium; designated Cell-CM) was collected. Neat SFM, i.e., without incubation in the presence of cells, was used as a control.

### Cell viability assay

To evaluate the sensitivity to adenoviruses of PSCs, cells of the hPSC-5 and hPSC-14 lines were seeded in 96-well plates at a density of 3 × 10^3^ and 4 × 10^3^ cells/well, respectively; the plates then were incubated for 24 h before viral infection. Culture medium was replaced with PDAC-CM or SFM at the same time as the cells were infected with Ad5, OBP-301, or OBP-702; infection with each virus was performed at multiplicities of infection (MOIs) of 0, 1, 5, 10, 50, and 100 plaque-forming units (PFU) per cell. Cell viability was determined at 72 h (OBP-301, OBP-702) or 96 h (Ad5) after virus infection using the Cell Proliferation Kit II (Roche Molecular Biochemicals, Indianapolis, IN, USA) according to the manufacturer’s protocol.

### Western blot analysis

hPSC-5 and hPSC-14 cells were seeded in a 100-mm dish at a density of 5 × 10^5^ cells/dish, and cells were incubated for 24 h before virus infection. Culture medium was replaced with PDAC-CM or SFM at the same time as the cells were infected with Ad5, OBP-301, or OBP-702; infection with each virus was performed at MOIs of 0, 10, 25, and 100 PFU/cell. The infected cells were incubated for 48 h (Ad5) or 72 h (OBP-301 and OBP-702). Whole-cell lysates then were prepared using a lysis buffer [50 mM Tris-HCl (pH 7.4), 150 mM NaCl, 1% Triton X-100] containing a protease inhibitor cocktail (Complete Mini; Roche, Indianapolis, IN, USA). The proteins in each lysate were separated by electrophoresis on 6% to 15% sodium dodecyl sulfate-polyacrylamide gels and transferred to polyvinylidene difluoride membranes (Hybond-P; GE Health Care, Buckinghamshire, UK). The resulting blots were blocked with Blocking One reagent (Nacalai Tesque, Kyoto, Japan) at room temperature (RT) for 30 min prior to sequential hybridization with the primary and secondary antibodies. The primary antibodies were as follows: mouse anti-Ad5 E1A monoclonal antibody (mAb) (#554155; BD PharMingen, Franklin Lakes, NJ, USA), mouse anti-p53 mAb (#18032; Cell Signaling Technology, Danvers, MA, USA), rabbit anti-poly(ADP-ribose) polymerase (PARP) polyclonal antibody (#9542; Cell Signaling Technology), and mouse anti-β-Actin mAb (#A5441; Sigma-Aldrich, St. Louis, MO, USA). The secondary antibodies were as follows: horseradish peroxidase-conjugated antibodies against mouse immunoglobulin G (IgG) (#NA931; GE Healthcare) or against rabbit IgG (#NA934; GE Healthcare). Immunoreactive bands on the blots were visualized using Enhanced Chemoluminescence substrates (ECL Prime; GE Healthcare) according to the manufacturer’s instructions.

### Real-time polymerase chain reaction (PCR) analysis

hPSC-5 cells were seeded in a 25 T flask at a density of 2.5 × 10^5^ cells/flask and grown for 24 h before viral infection. The culture medium then was replaced with 5 mL of PDAC-CM or SFM at the same time as the cells were infected with Ad5, OBP-301, or OBP-702 at an MOI of 25. The cells were harvested after 2 h of virus infection, and then subjected to DNA purification using the QIAmp DNA mini Kit (Qiagen, Hilden, Germany) according to the manufacturer’s protocol. *E1A* copy number was determined by quantitative real-time PCR using TaqMan real time PCR System (Applied Biosystems, Carlsbad, CA, USA).

### GFP expression assay

hPSC-5 cells were seeded in 24-well plates at a density of 2 × 10^4^ cells/well and grown for 24 h before virus infection. Culture medium was changed to CM derived from human normal and cancer cells or SFM as a control at the same time as the cells were infected with Ad-GFP or OBP-401 at an MOI of 100. Infected cells were incubated for 48 h. The cells then were fixed with 4% paraformaldehyde (PFA), permeabilized with methanol, and stained with 4’,6-diamidino-2-phenylindole (DAPI) to identify nuclei. For each well, five randomly selected fields were photographed using an IX83 fluorescence microscope (Olympus, Tokyo, Japan), and GFP intensities were calculated using ImageJ software.

### Flow cytometry

hPSC-5 and hPSC-14 cells were seeded in a 25 T flask at a density of 2.5 × 10^5^ cells/flask and grown for 24 h before treatment. The culture medium then was replaced with 5 mL of PDAC-CM or SFM and incubated for 48 h. To analyze the expression of coxsackie virus and adenovirus receptor (CAR) and integrins αvβ3 and αvβ5, cells were incubated with mouse anti-CAR monoclonal antibody (mAb) (RmcB; Upstate, Lake Placid, NY, USA), rabbit anti-integrin αvβ3 polyclonal antibody (pAb) (bs-1310R; Bioss Antibodies, Woburn, MA, USA), and rabbit anti- integrin αvβ5 pAb (bs-1356R; Bioss Antibodies) or isotype control IgG for 60 min on ice. The cells then were labeled with fluorescein isothiocyanate (FITC)-conjugated rabbit anti-mouse IgG secondary antibody (A16161; Invitrogen, Carlsbad, CA, USA) or Alexa Fluor 647-conjugated goat anti-rabbit IgG secondary antibody (A-21245; Invitrogen) for 30 min and then analyzed using FACS Lyric (BD Biosciences).

### Macropinocytosis assay

Cells were seeded in 8-well chambers at a density of 9 × 10^3^ cells/well and incubated for 24 h before treatment. Cells then were pretreated with 5-(N-ethyl-N-isopropyl) amiloride (EIPA, a known inhibitor of macropinocytosis; 100 μM) or dimethyl sulfoxide (DMSO; vehicle control) for 1 h before incubation in PDAC-CM or SFM for 2 h. Macropinocytic uptake by the cells then was assessed as follows: The medium was replaced with 70-kDa tetramethylrhodamine (TMR)-conjugated dextran (Invitrogen) formulated in SFM to a final concentration of 0.5 mg/mL, and the plate was incubated for 30 min. Plates then were placed on ice and rinsed five times with ice-cold PBS, at which point the cells were fixed in 4% PFA and stained with DAPI. Fluorescence images were obtained using an IX83 fluorescence microscope. The dextran index was calculated as described previously [23], using ImageJ software to determine the total particle area per cell.

### In vitro 3D culture model with PDAC cells and PSCs

The in vitro 3D culture model, which was used to mimic PDAC tumors with desmoplastic stromata, was conducted as reported previously [24]. Briefly, hPSC-4 and hPSC-15 cells were incubated (gentle rocking, RT, 30 min) in Tris-buffered saline (pH 7.4) containing 0.04 mg/mL fibronectin (Sigma-Aldrich) and 0.04 mg/mL gelatin (Wako Pure Chemicals, Osaka, Japan). PSCs then were centrifuged and re-suspended in the DMEM/Ham’s F-12 mixture. Separately, BxPC-3 cells also were suspended in the DMEM/Ham’s F-12 mixture. The two cell suspensions (one PSC, one PDAC) were mixed (as indicated below) to yield a final volume of 500 μL. For 3D PDAC tissues generated by the co-culture of PSCs and PDAC cells, the seeding ratio of PSCs and PDAC cells was 100:1 (2.5 × 10^5^ PSCs and 2.5 × 10^3^ BxPC-3 cells). The cell mixture was seeded on cell culture inserts for 24-well plates (0.4-μm pores, transparent; BD Falcon/Corning, Corning, NY, USA) that had been pre-coated by incubation for >30 min with 0.12 mg/mL fibronectin. The culture medium in the inserts was replaced on Day 2. On Day 4, the culture medium was replaced again, and the cells were infected with OBP-301 or OBP-702, provided at each of three dose levels: 1 × 10^6^ PFU (low MOI), 5 × 10^6^ PFU (moderate MOI), and 1 × 10^7^ PFU (high MOI). On Day 7 (72 h after virus infection), the 3D tissues were fixed (RT, 5 min) with 4% PFA in PBS and permeabilized (RT, 20 min) with 0.2% Triton X-100 in PBS. The fixed and permeabilized 3D tissues then were blocked with Blocking One reagent and incubated (4 °C, overnight) with primary antibodies. The primary antibodies used in this experiment were as follows: mouse anti-pan-keratin mAb (#4545; Cell Signaling Technology) and rabbit anti-p53 mAb (#2527; Cell Signaling Technology). After three rounds of washing with PBS, the 3D tissues were incubated with Alexa Fluor-labeled secondary antibodies (Molecular Probes, Eugene, OR, USA). The 3D tissues subsequently were counter-stained with Hoechst 33342 (Thermo Fisher Scientific, Waltham, MA, USA; to visualize nuclear DNA) and washed with PBS. The culture insert membranes then were carefully excised using a scalpel and mounted on coverslips using fluorescent mounting medium (Dako, Santa Clara, CA, USA). Samples were observed under an LSM780 confocal laser microscope (Zeiss, Oberkochen, Germany). To detect apoptotic cells, the Click-it TUNEL Alexa Fluor Imaging Assay Kit (Invitrogen) was used (per the manufacturer’s protocol) after fixation and permeabilization. All other steps were performed as described above.

### In vivo subcutaneous BxPC-3 xenograft tumor model

All animal experimental protocols were approved by the Ethics Review Committee for Animal Experimentation of Okayama University School of Medicine (Approval No. OKU-2018319). To evaluate the role of PSCs in the growth of PDAC tumors, BxPC-3 cells (1 × 10^6^ cells/site), alone or in combination with hPSC-14 cells (9 × 10^6^ cells/site), were subcutaneously inoculated into the right flank of 6-week-old female BALB/c-nu/nu mice (CLEA Japan, Tokyo, Japan) (n = 4). To compare the therapeutic potential of OBP-301 and OBP-702 against BxPC-3 tumors grown in combination with PSCs, BxPC-3 cells (1 × 10^6^ cells/site) and hPSC-14 cells (9 × 10^6^ cells/site) were subcutaneously co-inoculated into the right flank of 6-week-old female BALB/c-nu/nu mice. When tumors had achieved volumes of ~100 mm^3^, tumor-bearing mice were randomized and assigned to three groups (n = 10). A 50-μL volume of solution containing OBP-301 or OBP-702 at a dose of 1 × 10^8^ PFU, or PBS (mock control) was injected intratumorally (the nominal Day 0); dosing with the respective test or control article was repeated on Days 2 and 4, for a total of three injections. Tumor volumes were measured every 2–3 days throughout the in-life interval. Specifically, tumor volumes were determined by measuring the perpendicular axes of each tumor and using the values in the formula for the volume of an ellipsoid, as follows: tumor volume (mm^3^) = *a* × *b*^2^ × 0.5, where *a* and b represent the longer and shorter perpendicular axes (respectively).

### Immunofluorescent staining of tumors treated with oncolytic adenoviruses

Following euthanasia of the mice, tumors were excised, fixed in 10% neutral buffered formalin, and embedded in paraffin blocks. Sections (4-μm thick) were incubated first with mouse anti-pan-keratin mAb and rabbit anti-p53 mAb or rabbit anti-Ad5-hexon mAb (#ab316852; Abcam, Cambridge, UK), and then with the respective Alexa Fluor-labeled secondary antibodies. Finally, sections were counterstained using Nucblue (Thermo Fisher Scientific; providing staining of nuclear DNA) and observed using an IX83 fluorescence microscope.

### Statistical analysis

Where appropriate, data are expressed as mean ± standard deviation (SD). For comparisons between 2 groups, statistical significance was determined using a two-tailed, non-paired Student’s *t* test. For comparisons of more than 2 groups, statistical significance was determined using a two-tailed one-way ANOVA followed by a Tukey’s multiple comparison procedure. Statistical significance was defined as a *P* value less than 0.05.

## Results

### Enhancement of sensitivity to adenovirus in PDAC-associated PSCs

**Fig. 1:**
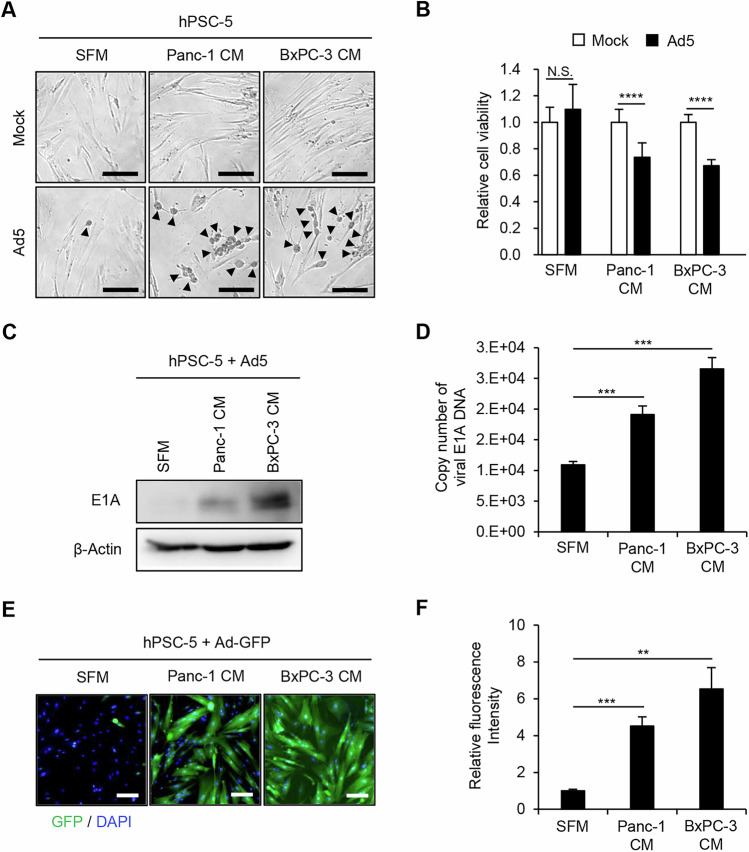
Potentiation of sensitivity to adenoviruses in PSCs incubated with PDAC-CM. **A** Representative images of morphological changes, including the rounding of the cells (black arrowheads), in hPSC-5 cells infected with Ad5 (MOI 100) for 96 h in the presence of SFM or PDAC-CM. Scale bars: 200 μm. **B** hPSC-5 cells were infected with Ad5 (MOI 100) for 96 h in the presence of SFM or PDAC-CM, and cell viability was quantified using the XTT assay. Cell viability was calculated relative to that of the mock-infected group, which was set at 1.0. Cell viability data are expressed as mean ± SD (n = 5). **C** hPSC-5 cells were infected with Ad5 (MOI 25) for 48 h in the presence of SFM or PDAC-CM. Cell lysates were subjected to western blot analysis for adenoviral E1A protein. β-Actin was assessed as a loading control. **D** Quantitative measurement of copy number of viral E1A DNA in hPSC-5 cells infected with Ad5 (MOI 25) for 2 h in the presence of SFM or PDAC-CM. Data are expressed as mean ± SD (n = 3). **E** Representative images of GFP fluorescence in hPSC-5 cells after infection with Ad-GFP in the presence of SFM or PDAC-CM. Green and blue color indicate fluorescence of GFP and DAPI (nuclear DNA), respectively. Scale bars: 200 μm. **F** Relative fluorescence intensity of GFP was analyzed using ImageJ software. Data are expressed as mean ± SD (n = 3). The statistical significance of differences between two groups was determined using the two-tailed, non-paired Student’s *t*-test. N.S., not significant (P ≥ 0.05); **, *P* < 0.01; ***, *P* < 0.001; ****, *P* < 0.0001 (vs Mock or SFM).

To investigate whether PDAC-associated PSCs are sensitive to adenovirus, hPSC-5 cells were infected with Ad5 (MOI 100) for 96 h in the presence of SFM or PDAC-CM (consisting of BxPC-3-CM or Panc-1-CM). Morphological changes, notably including rounding of the cells, were observed in Ad5-infected hPSC-5 cells incubated in the presence of PDAC-CM; these changes were stronger in the presence of PDAC-CM than in the presence of SFM (Fig. 1A). To evaluate the cytopathic effect of Ad5 in PDAC-associated PSCs, the XTT (sodium 3´-[1- (phenylaminocarbonyl)- 3,4- tetrazolium]-bis (4-methoxy6-nitro) benzene sulfonic acid hydrate) assay was used to assess the viability of hPSC-5 cells following infection with Ad5 for 96 h in the presence of SFM or PDAC-CM. The XTT assay demonstrated that Ad5 infection resulted in significantly greater attenuation of the viability of PDAC-CM-incubated hPSC-5 cells than of SFM-incubated hPSC-5 cells (Fig. 1B). These results suggested that PDAC-associated PSCs are more sensitive to adenovirus than are non-activated PSCs.

To further confirm the enhancement of sensitivity to adenovirus in PDAC-associated PSCs, SFM- and PDAC-CM-incubated hPSC-5 cells were infected with Ad5 (MOI 25). Adenoviral E1A protein level and viral DNA copy number were analyzed by western blot and real-time PCR analyses, respectively. The immunoblotting showed that PDAC-CM-incubated hPSC-5 cells exhibited increased levels of viral E1A protein compared to SFM-incubated cells (Fig. 1C). Quantitative real-time PCR demonstrated that the copy number of the *E1A* gene was significantly higher in PDAC-CM-incubated hPSC-5 cells than in SFM-incubated cells (Fig. 1D). Next, to evaluate whether virus entry is increased in PDAC-associated PSCs, we employed a replication-deficient, GFP-expressing adenovirus (Ad-GFP); notably, Ad-GFP-mediated GFP production permits the detection of adenovirus-sensitive cells as GFP-positive cells. PDAC-CM-incubated hPSC-5 cells showed significantly higher GFP accumulation than did SFM-incubated cells (Fig. 1E, F). Together, these results indicated that PDAC-associated PSCs are more sensitive to adenovirus than are control PSCs, suggesting that this increased sensitivity to infection reflects enhancement of virus entry.

To evaluate whether high virus sensitivity depends on the increased expression of adenovirus receptors, including coxsackie virus and adenovirus receptor (CAR) and integrins αvβ3 and αvβ5, SFM- and PDAC-CM-incubated hPSC-5 and hPSC-14 cells were analyzed by flow cytometry. The expression of CAR and integrins αvβ3 and αvβ5 was not increased in PDAC-CM-incubated hPSC-5 and hPSC-14 cells (Fig. S1). These results indicated that PDAC-associated PSCs exhibit high virus sensitivity independent of adenovirus receptors.

### Enhancement of virus sensitivity in PDAC-associated PSCs is associated with macropinocytosis activation

**Fig. 2:**
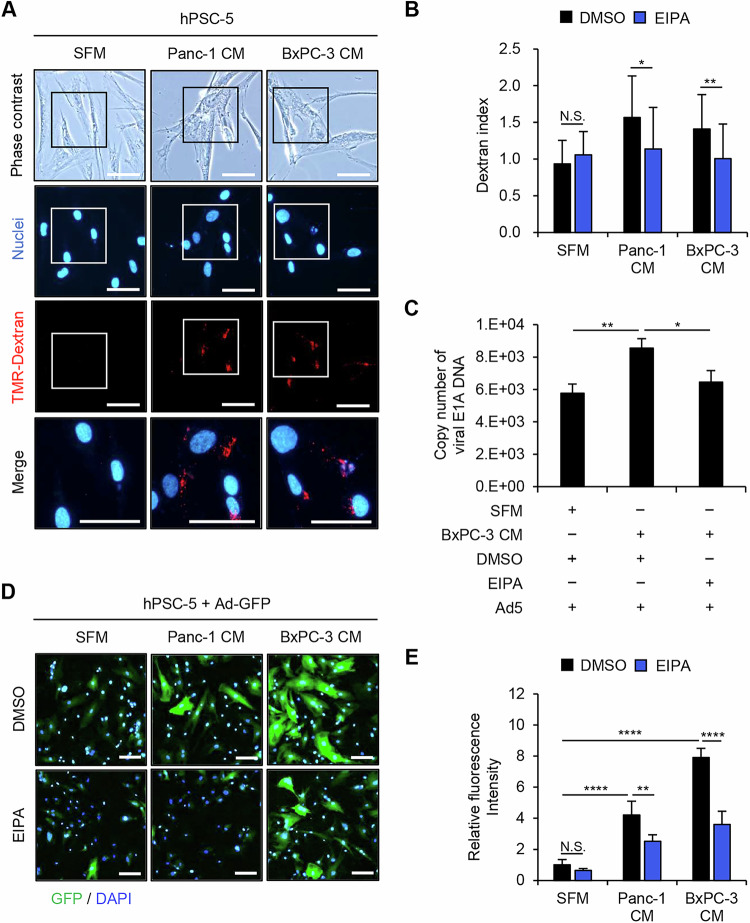
Macropinocytosis-mediated potentiation of sensitivity to adenoviruses in PSCs incubated with PDAC-CM. **A** Representative images of TMR-dextran accumulation by hPSC-5 cells incubated in the presence of SFM or PDAC-CM. Scale bars: 200 μm. **B** hPSC-5 cells were treated with DMSO or EIPA in the presence of TMR-dextran in SFM or PDAC-CM, and the dextran index was calculated using ImageJ software. DMSO was used as the vehicle control. **C** Quantitative measurement of copy number of viral *E1A* DNA in hPSC-5 cells treated with DMSO or EIPA and infected with Ad5 (MOI 25) for 2 h in the presence of SFM or PDAC-CM. **D** Representative images of GFP expression in hPSC-5 cells treated with DMSO or EIPA and infected with Ad-GFP (MOI 100) for 48 h in the presence of SFM or PDAC-CM. Scale bars: 200 μm. **E** Relative fluorescence intensity of GFP was analyzed using ImageJ software. Data are expressed as mean ± SD (n = 3). The statistical significance of differences between two groups or three groups was determined using the two-tailed, non-paired Student’s *t*-test or two-tailed one-way ANOVA followed by Tukey’s comparison tests, respectively. N.S., not significant; *, *P* < 0.05; **, *P* < 0.01; ****, *P* < 0.0001 (vs DMSO or SFM).

Macropinocytosis has been shown to serve as a receptor-independent route of virus entry for several types of viruses, including Ad5 [20]. Given that macropinocytosis plays an important role in the uptake of extracellular particles [20], we analyzed the accumulation of fluorescent tag-conjugated dextran (TMR-dextran) by SFM- and PDAC-CM-incubated hPSC-5 cells. PDAC-CM-incubated hPSC-5 cells showed increased accumulation of TMR-dextran compared to SFM-incubated cells (Fig. 2A). To evaluate the role of macropinocytosis in the accumulation of TMR-dextran in PDAC-associated PSCs, SFM- and PDAC-CM-incubated hPSC-5 cells were exposed to EIPA, a selective inhibitor of macropinocytosis; DMSO was employed as a vehicle control. Compared to DMSO exposure, EIPA exposure significantly attenuated the accumulation of TMR-dextran in PDAC-CM-incubated hPSC-5 cells (Fig. 2B). These results suggested the potentiation of macropinocytosis in PDAC-associated PSCs (compared to control PSCs).

To evaluate whether macropinocytosis activation is involved in the sensitivity to adenovirus of PDAC-associated PSCs, SFM- and PDAC-CM-incubated hPSC-5 cells were infected with Ad5 in the presence of EIPA or DMSO. Compared to DMSO exposure, EIPA exposure resulted in a significant decrease in the copy number of viral *E1A* in BxPC-3-CM-incubated hPSC-5 cells (Fig. 2C). Next, SFM- and PDAC-CM-incubated hPSC-5 cells were infected with Ad-GFP in the presence or absence of EIPA. Notably, PDAC-CM-incubated hPSC-5 cells showed a significantly higher accumulation of GFP compared to SFM-incubated cells (Fig. 2D, E). Moreover, compared to DMSO exposure, EIPA exposure resulted in a significant decrease in the accumulation of GFP in PDAC-CM-incubated hPSC-5 cells (Fig. 2D, E). Together, these data suggested that induction of macropinocytosis contributes to the enhancement of sensitivity to adenovirus in PDAC-activated PSCs.

### Enhancement of sensitivity to oncolytic adenoviruses in PDAC-associated PSCs with macropinocytosis activation

**Fig. 3:**
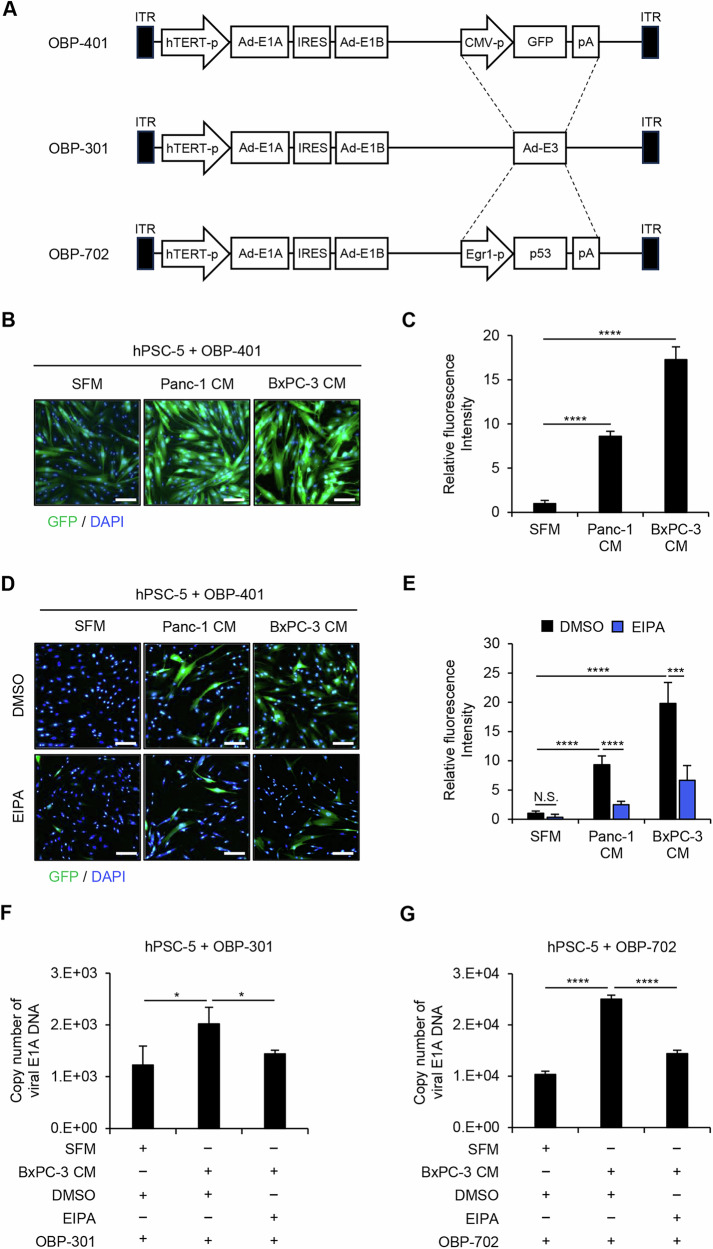
Macropinocytosis-mediated potentiation of sensitivity to oncolytic adenoviruses in PSCs incubated with PDAC-CM. **A** Schematic diagrams of structures of the oncolytic adenoviruses. OBP-301 is a telomerase-specific, replication-competent oncolytic adenovirus in which the hTERT promoter drives expression of the adenoviral E1A and E1B genes, which are linked by an IRES. OBP-401 is a GFP-expressing OBP-301 variant generated by inserting the CMV promoter-driven *GFP* gene expression cassette into the E3 region of OBP-301. OBP-702 is a p53-armed OBP-301 variant generated by inserting the Egr1 promoter-driven human wild-type p53 gene expression cassette into the E3 region of OBP-301. **B** Representative images of GFP fluorescence in hPSC-5 cells following infection with OBP-401 (MOI 100) for 48 h in the presence of SFM or PDAC-CM. Scale bars: 200 μm. **C** Relative fluorescence intensity of GFP was analyzed by ImageJ software. **D** Representative images of GFP fluorescence in hPSC-5 cells treated with DMSO or EIPA and infected with OBP-401 (MOI 100) for 48 h in the presence of SFM or PDAC-CM. Scale bars: 200 μm. **E** Relative fluorescence intensity of GFP was analyzed using ImageJ software. **F**, **G** Quantitative measurement of copy number of viral *E1A* DNA in hPSC-5 cells treated with DMSO or EIPA and infected with OBP-301 (MOI 25) (F) or OBP-702 (MOI 25) (G) for 2 h in the presence of SFM or PDAC-CM. Data are expressed as mean ± SD (n = 3). The statistical significance of differences between two groups or three groups was determined using the two-tailed, non-paired Student’s *t*-test or two-tailed one-way ANOVA followed by Tukey’s comparison tests, respectively. N.S., not significant; *, *P* < 0.05; ***, *P* < 0.001; ****, *P* < 0.0001.

We previously demonstrated the diagnostic potential of OBP-401 (Fig. 3A), a GFP-expressing, telomerase-specific, replication-competent oncolytic adenovirus; specifically, OBP-401 permitted the detection of oncolytic adenovirus-sensitive cells as GFP-positive cells [22]. To evaluate the sensitivity to oncolytic adenoviruses of PDAC-associated PSCs, SFM- and PDAC-CM-incubated hPSC-5 cells were infected with the GFP-expressing OBP-401 adenovirus for 48 h, at which point the accumulation of GFP was analyzed by fluorescence microscopy. PDAC-CM-incubated hPSC-5 cells showed a significantly greater accumulation of GFP compared to SFM-incubated cells (Fig. 3B, C). To investigate the role of macropinocytosis in the enhancement of sensitivity to OBP-401 in PDAC-associated PSCs, SFM- and PDAC-CM-incubated hPSC-5 cells were infected with OBP-401 in the presence of EIPA or DMSO. Compared to DMSO exposure, EIPA exposure resulted in a significant decrease in the accumulation of GFP in PDAC-CM-incubated hPSC-5 cells (Fig. 3D, E). These data suggested that the activation of macropinocytosis contributes to the enhancement of sensitivity to oncolytic adenoviruses seen in PDAC-associated PSCs.

To investigate the role of CM derived from human cancer cells in the sensitivity to OBP-401-mediated GFP expression in PSCs, hPSC-5 cells incubated in the CM obtained from human cancer cells (H1299, A549, NUGC4, or MDA-MB-231) or from human normal fibroblasts (WI38) were infected with OBP-401 for 48 h. Human cancer cell-CM-incubated hPSC-5 cells showed a significantly higher accumulation of GFP than SFM-incubated cells (Fig. S2). In contrast, WI38-CM or fresh culture medium containing 10% FBS did not enhance the accumulation of GFP in OBP-401-infected hPSC-5 cells (Fig. S2). These results suggested that tumor cell-associated PSCs are sensitive to oncolytic adenoviruses.

We recently demonstrated that telomerase-specific, replication-competent oncolytic adenoviruses OBP-301 and p53-armed OBP-702 (Fig. 3A) have therapeutic potential against human PDAC cells [19]. To investigate the role of macropinocytosis in the enhancement of sensitivity to oncolytic adenoviruses in PDAC-associated PSCs, SFM- and PDAC-CM-incubated hPSC-5 cells were infected with OBP-301 or OBP-702 for 2 h in the presence or absence of EIPA. The *E1A* DNA copy number then was analyzed by real-time PCR analysis. Quantitative real-time PCR demonstrated that the copy number of *E1A* DNA was significantly higher in OBP-301- and OBP-702-infected hPSC-5 cells incubated in the presence of PDAC-CM (compared to infected cells incubated in the presence of SFM) (Fig. 3F, G). Moreover, compared to the control, EIPA exposure significantly attenuated the increase in viral *E1A* copy number otherwise seen in PDAC-CM-incubated hPSC-5 cells infected with OBP-301 or OBP-702 (Fig. 3F, G). Together, these results suggested that PDAC-associated PSCs are more sensitive to oncolytic adenoviruses than are control PSCs.

### Decreased viability of PDAC-associated PSCs following OBP-702 infection results from p53-mediated induction of apoptosis

**Fig. 4:**
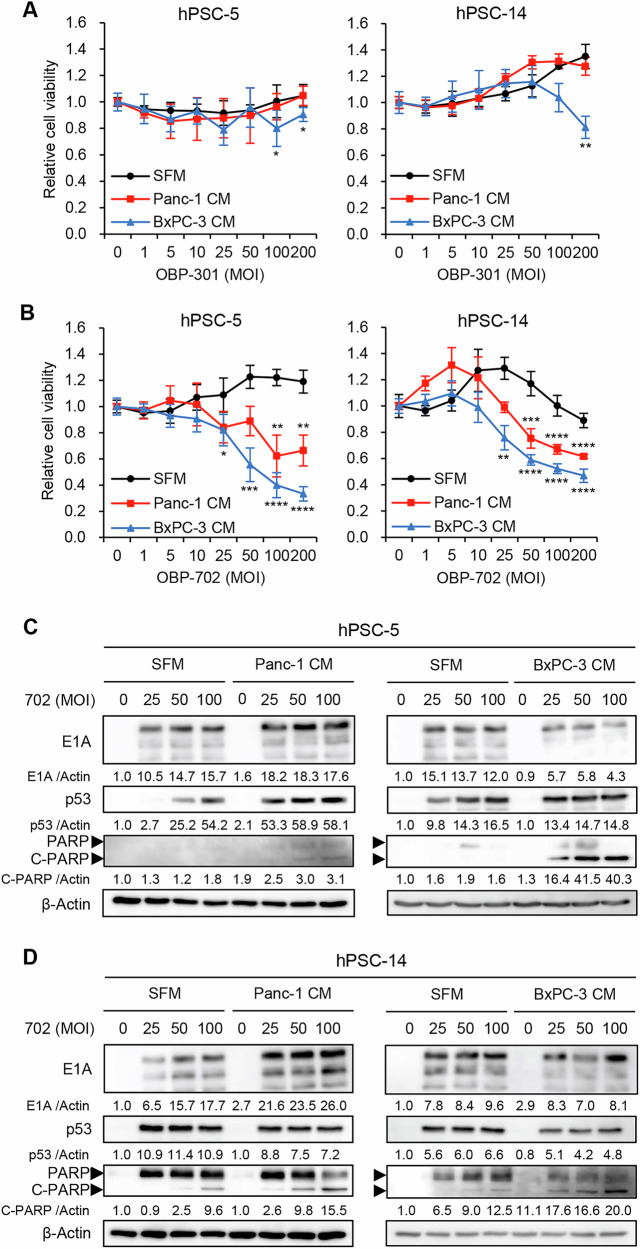
Cytopathic effect and apoptosis induction by OBP-702 in PSCs incubated with PDAC-CM. **A**, **B** hPSC-5 and hPSC-14 cells were infected with OBP-301 (**A**) or OBP-702 (**B**) at the indicated MOIs for 72 h in the presence of SFM or PDAC-CM, and cell viability was quantified using the XTT assay. Cell viability was calculated relative to that of the mock-infected group (MOI 0), which was set at 1.0. Cell viability data are expressed as mean ± SD (n = 5). The statistical significance of differences between two groups was determined using the two-tailed, non-paired Student’s *t*-test. *, *P* < 0.05; **, *P* < 0.01; ***, *P* < 0.001; ****, *P* < 0.0001 (vs MOI 0). hPSC-5 (**C**) and hPSC-14 (**D**) cells were infected with OBP-702 at the indicated MOIs for 72 h in the presence of SFM or PDAC-CM. Cell lysates were subjected to western blot analysis for adenoviral E1A, p53, PARP, and cleaved PARP (C-PARP) proteins. β-Actin was assayed as a loading control. The expression level of each protein was calculated relative to its expression in mock-infected cells incubated in SFM, whose expression level was designated as 1.0.

To investigate whether oncolytic adenoviruses have therapeutic potential against PDAC-associated PSCs, the XTT assay was used to assess the viabilities of hPSC-5 and hPSC-14 cells following a 72-h infection with OBP-301 or OBP-702 in the presence of SFM or PDAC-CM. The XTT assay demonstrated that OBP-301 infection at high MOIs decreased the viability of hPSC-5 and hPSC-14 cells incubated with BxPC-3-CM, but not that of cells incubated with Panc-1-CM (Fig. 4A). In contrast, OBP-702 infection at moderate and high MOIs significantly decreased the viabilities of hPSC-5 and hPSC-14 cells incubated with either Panc-1-CM or BxPC-3-CM (Fig. 4B). Control experiments demonstrated that non-activated PSCs were resistant to OBP-301 and OBP-702 compared to the PDAC-associated PSCs (Fig. 4A, B). These results suggested that p53-armed OBP-702 is superior to OBP-301 in cytopathic activity against PDAC-associated PSCs.

In previous work, we demonstrated that OBP-702 has therapeutic potential, based on the ability of this oncolytic adenovirus to induce p53 expression and apoptosis in human PDAC cells [19]. To evaluate whether OBP-702 induces p53 expression and apoptosis in PDAC-associated PSCs, hPSC-5 and hPSC-14 cells were infected with OBP-702 for 72 h. The levels of E1A, p53, and PARP proteins then were determined by western blotting; in this context, PARP cleavage is a marker of apoptosis. This analysis demonstrated that OBP-702-infected, Panc-1-CM-incubated hPSC-5 cells accumulated the E1A, p53, and cleaved PARP (C-PARP) proteins to higher levels than those seen in mock-infected cells incubated in SFM (Fig. 4C). BxPC-3-CM-incubated hPSC-5 cells showed increased expression of C-PARP after OBP-702 infection compared with SFM-incubated cells, although the expression level of E1A and p53 proteins was not increased in BxPC-3-CM-incubated cells (Fig. 4C). The infection of hPSC-14 cells with OBP-702 at high MOIs resulted in the accumulation of the E1A and C-PARP proteins to higher levels in Panc-1-CM-incubated cells than in SFM-incubated cells (Fig. 4D). BxPC-3-CM-incubated hPSC-14 cells showed increased expression of C-PARP after OBP-702 infection compared to SFM-incubated cells, although the expression level of E1A protein was not increased in BxPC-3-CM-incubated cells (Fig. 4D). The expression level of p53 protein was slightly decreased in PDAC-CM-incubated hPSC-14 cells (Fig. 4D). Together, these results suggested that p53-armed OBP-702 has the therapeutic potential to induce apoptosis in PDAC-associated PSCs. Given that a previous report showed that E1A expression decreases during the late phase of adenovirus infection [25], the increased virus sensitivity observed in OBP-702 infected, BxPC-3-CM-incubated PSCs suggests that these cells may be at the late phase of infection.

### Suppression of PDAC cells and PDAC-associated PSCs by OBP-702 in a 3D culture model

**Fig. 5:**
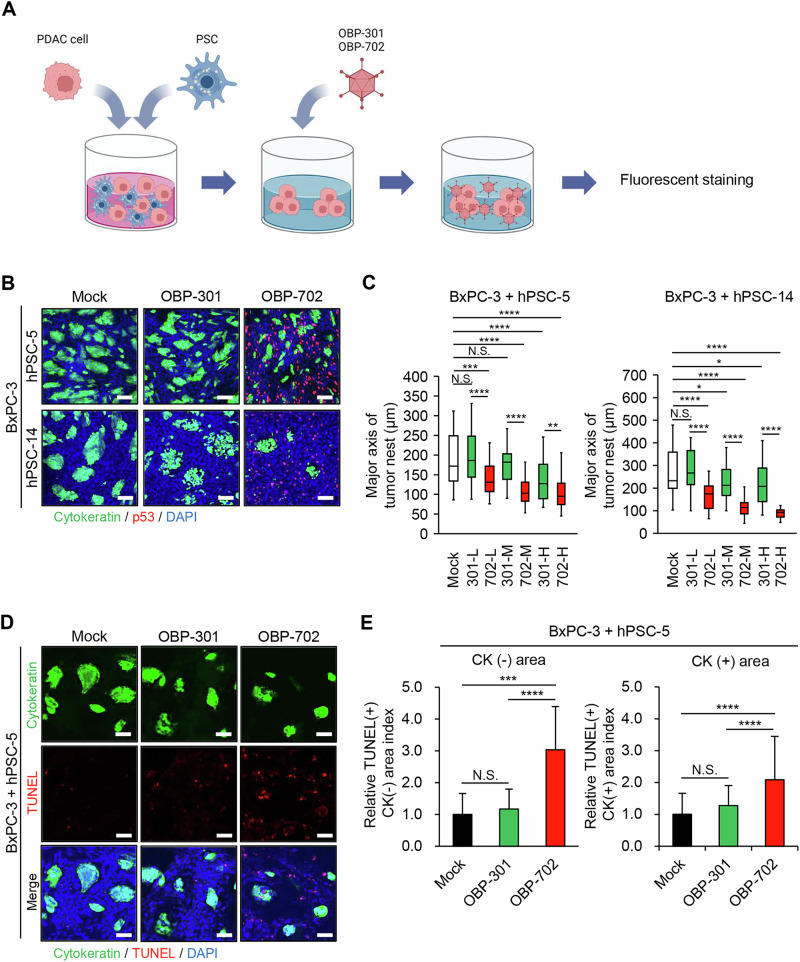
Attenuation of PDAC tumor nests by oncolytic adenoviruses in 3D culture models with PDAC cells and PSCs. **A** Schema of experimental protocol using a 3D culture model. PDAC cells and PSCs were co-cultured to generate the 3D tissues. The 3D tissues then were infected at low, moderate, and high MOIs of OBP-301 or OBP-702 for 72 h. Subsequently, the infected 3D tissues were stained by immunohistochemistry. **B** Representative images of staining for cytokeratin and p53 in the 3D tissues obtained with BxPC-3 cells and hPSC-5 or hPSC-14 cells following infection with mock (MOI 0), OBP-301, or OBP-702 for 72 h. Scale bars: 200 μm. **C** Quantitative measurement of the major axis of tumor nests in the 3D tissues obtained with BxPC-3 cells and hPSC-5 or hPSC-14 cells. **D** Representative images of staining for cytokeratin and by TUNEL in the 3D tissues obtained with BxPC-3 cells and hPSC-5 cells following infection with mock (MOI 0), OBP-301, or OBP-702 for 72 h. Scale bars: 100 μm. **E** Quantitative measurement of relative TUNEL-positive area index in cytokeratin-negative and cytokeratin-positive areas in the 3D tissues obtained with BxPC-3 and hPSC-5 cells after infection with mock (MOI 0), OBP-301, or OBP-702 for 72 h. DAPI was used for nuclear counter-staining. Data are expressed as mean ± SD (n = 3). The statistical significance of differences between two groups or three groups was determined using the two-tailed, non-paired Student’s *t*-test or two-tailed one-way ANOVA followed by Tukey’s comparison test, respectively. N.S., not significant; *, *P* < 0.05; **, *P* < 0.01; ***, *P* < 0.001; ****, *P* < 0.0001 (vs Mock or OBP-301).

To investigate whether OBP-702 exerts in vitro antitumor effects against PDAC tumors containing PSCs, we employed a 3D culture model with PDAC cells and PSCs, a system that mimics PDAC tumors with fibrotic stromata that are composed primarily of PDAC-associated PSCs [24]. hPSC-5 or hPSC-14 cells were co-cultured with BxPC-3 cells to generate the 3D tissues, followed by infection for 72 h with low, moderate, and high MOIs of OBP-301 or OBP-702. (Fig. 5A). The 3D tissues then were stained immunohistochemically for cytokeratin and p53 to analyze the size of cytokeratin (+) PDAC tumor nests and p53 accumulation in cytokeratin (-) PDAC-associated PSCs (Fig. 5A). Although BxPC-3 cells harbor the Y220C mutant p53 protein, PDAC tumor nests showed low expression level of p53 protein (Fig. S3). This analysis demonstrated that (compared to uninfected cultures) cultures infected with OBP-702 exhibited a strong decrease in the area of cytokeratin (+) PDAC tumor nests along with an increase in the area of p53-staining cytokeratin (-) PDAC-associated PSCs (Fig. 5B). In contrast, OBP-301 infection resulted in a nominal decrease compared to uninfected cultures in the area of cytokeratin (+) PDAC tumor nests without an apparent increase in the area of p53-staining cytokeratin (-) PDAC-associated PSCs (Fig. 5B). For infection at the low MOI, OBP-702 provided a significant decrease (compared to the uninfected control) in the size of cytokeratin (+) PDAC tumor nests, whereas OBP-301 did not (Fig. 5C). For infection at moderate and high MOIs, both OBP-702 and OBP-301 provided significant decreases (compared to the uninfected control) in the size of cytokeratin (+) PDAC tumor nests, although the effect was nominally greater with OBP-702 than with OBP-301 (Fig. 5C). To further evaluate whether oncolytic adenoviruses induce apoptosis in vitro (in the 3D tissues), virus-infected 3D tissues were analyzed by the TUNEL (terminal deoxynucleotidyl transferase dUTP nick end labeling) assay. This assay demonstrated that (compared to uninfected controls) OBP-702 significantly increased the number of TUNEL (+) cells in the cytokeratin (-) PDAC-associated PSCs and cytokeratin (+) PDAC tumor nests, whereas OBP-301 did not (Fig. 5D, E). Together, these results suggested that OBP-702 has therapeutic potential for the elimination of both PDAC cells and PDAC-associated PSCs via induction of apoptosis.

### Inhibition of in vivo growth of PDAC tumors with stromata by OBP-702 in a subcutaneous xenograft tumor model

**Fig. 6:**
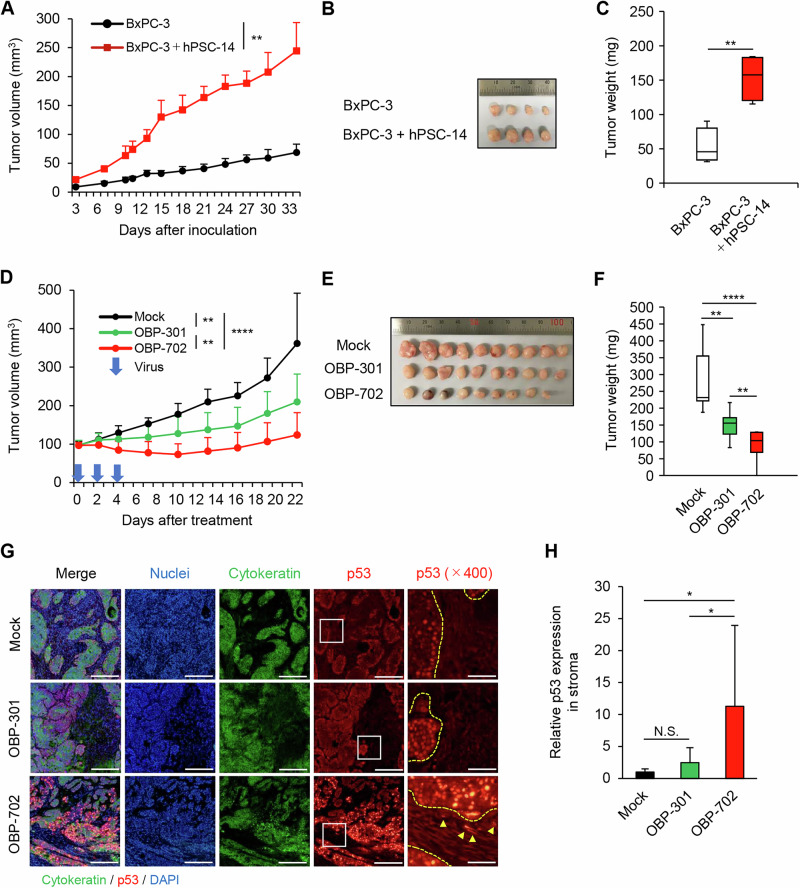
Attenuation of growth of PDAC + PSC tumors with stromal p53 expression by oncolytic adenoviruses. **A** BxPC-3 cells (1 × 10^6^ cells/site) and hPSC-14 cells (9 × 10^6^ cells/site) were co-inoculated subcutaneously in athymic nude mice. Tumor growth curves for BxPC-3 tumors (black line) or BxPC-3 + hPSC-14 tumors (red line). Data are expressed as mean ± SD (n = 4). Photographs of tumors (**B**) and plot of mean tumor weights (**C**) in mice with BxPC-3 tumors or BxPC-3 + hPSC-14 tumors. Box-and-whisker plots (in both **C**, **F**) show the median (central horizontal line), with boxes extending to the 25th and 75th quartiles; error bars extend to the minimum and maximum values. **D** BxPC-3 cells (1 × 10^6^ cells/site) and hPSC-14 cells (9 × 10^6^ cells/site) were co-inoculated subcutaneously in athymic nude mice. Tumor growth curves of mock- (MOI 0; black line), OBP-301- (green line), and OBP-702- (red line) infected tumors. Blue arrows indicate virus treatment. Data are expressed as mean ± SD (n = 10). Photographs of tumors (**E**) and plot of mean tumor weight (**F**) in mock- (PBS), OBP-301-, and OBP-702-infected tumors. **G** Representative images of immunohistochemical staining for cytokeratin and p53 in BxPC-3 + hPSC-5 tumors treated with mock (PBS), OBP-301, or OBP-702. DAPI was used for nuclear counter-staining. Yellow arrowheads represent p53-positive cells in the tumor stroma. Scale bars: 200 μm (left 4 columns) or 50 μm (right-most column; indicated as “× 400”). **H** Quantitative measurement of relative p53 staining in cytokeratin-negative stroma area of BxPC-3 + hPSC-5 tumors treated with mock, OBP-301, or OBP-702. Data are expressed as mean ± SD (n = 10). The statistical significance of differences between two groups or three groups was determined using the two-tailed, non-paired Student’s *t*-test or two-tailed one-way ANOVA followed by Tukey’s comparison test, respectively. N.S. not significant; *, *P* < 0.05; **, *P* < 0.01; ****, *P* < 0.0001.

We recently demonstrated the in vivo antitumor effect of OBP-301 and OBP-702 using a BxPC-3 xenograft tumor model [19]. Subcutaneous BxPC-3 xenograft tumor models demonstrated that OBP-301 and OBP-702 exhibited similar antitumor effects [19], suggesting that BxPC-3 xenograft tumor models are sensitive to both OBP-301 and OBP-702. As BxPC-3 CM increased the sensitivity to OBP-301 and OBP-702 in PDAC-associated PSCs (Fig. 4), BxPC-3 xenograft tumor models containing PSCs would be suitable for evaluating the effect of OBP-301 and OBP-702 against PDAC-associated PSCs. To develop PDAC tumors containing PSCs, BxPC-3 and hPSC-14 cells were subcutaneously co-inoculated into athymic nude mice. Co-inoculation of hPSC-14 cells and BxPC-3 cells resulted in significantly enhanced growth (as assessed by both volume and weight) of BxPC-3 tumors compared to that observed following inoculation of BxPC-3 cells alone (Fig. 6A–C). These results suggested that tumors composed of BxPC-3 and hPSC-14 show a phenotype of increased aggressiveness of growth compared to tumors derived from BxPC-3 alone.

To investigate the in vivo antitumor effects of oncolytic adenoviruses against PDAC tumors containing PSCs, mice harboring BxPC-3 + hPSC-14 tumors were administered (by intratumoral injection) OBP-301 or OBP-702 on Days 0, 2, and 4. Compared to mock treatment, intratumoral injection of OBP-301 or OBP-702 resulted in significant attenuation of the size (volume) and weight of BxPC-3 + hPSC-14 tumors (Fig. 6D-F). Moreover, treatment with OBP-702 resulted in significantly stronger antitumor effects than did OBP-301 (Fig. 6D-F). These results suggested that p53-armed OBP-702 is superior to OBP-301 in antitumor activity against PDAC + PSC tumors.

To evaluate whether OBP-702 induces p53 production in tumor and stromal areas of PDAC + PSC tumors, virus-treated BxPC-3 + hPSC-14 tumors recovered at necropsy were analyzed via immunohistochemical staining for cytokeratin and p53. This test demonstrated that, even in mock-treated tumors, the cytokeratin-positive region of the tumor exhibited staining for p53, indicating that BxPC-3-derived cells were producing (in vivo) the endogenous mutant p53 protein (Fig. 6G). However, intratumoral injection of the tumors with OBP-702 resulted in increased staining for p53 in the cytokeratin-positive region of the tumor compared to mock or OBP-301 treatment (Fig. 6G). Moreover, for the cytokeratin-negative stromal area of the tumor tissues, OBP-702 infection resulted in significantly increased staining for p53 compared to mock or OBP-301 treatment (Fig. 6H). To evaluate the infectivity of oncolytic adenoviruses in the stroma of BxPC-3 tumors, we investigated the distribution of oncolytic adenoviruses by immunohistochemistry for the Ad5-hexon protein. OBP-702-treated tumors, but not OBP-301-treated tumors, showed the presence of Ad5-hexon-positive cells in the tumor stroma (Fig. S4). Together, these results suggested that OBP-702 has the therapeutic potential to exert a strong in vivo antitumor effect by preventing the growth of PDAC cells and PDAC-activated PSCs via activation of p53.

## Discussion

Crosstalk between PDAC cells and PSCs is known to contribute to tumor progression [26]. Given the high clinical mortality rates associated with PDAC, novel therapeutic modalities must be developed to eliminate PDAC cells and PDAC-associated PSCs in patients. In the present study, we demonstrated that PDAC-associated PSCs are more sensitive to wild-type and oncolytic adenoviruses than are control PSCs, a difference that reflects the potentiation of macropinocytosis-mediated virus entry in PDAC-associated PSCs. Notably, the macropinocytosis inhibitor EIPA significantly attenuated the sensitivity to wild-type and oncolytic adenoviruses of PDAC-associated PSCs. We further observed that the p53-armed OBP-702 exhibits a stronger antitumor effect than a non-armed OBP-301; this difference appears to result from the p53-mediated induction of apoptosis, as demonstrated in vitro in a 3D culture model incorporating both PDAC cells and PSCs. Moreover, we showed that intratumoral injection of OBP-702 provides stronger in vivo anti-tumor activity against PDAC + PSC tumors than does the non-armed OBP-301 virus; again, this difference correlated with increased accumulation of p53, in this case in the tumor stroma. Our findings suggest that the increased sensitivity to oncolytic adenoviruses exhibited by PDAC-associated PSCs reflects increases in macropinocytosis-mediated virus entry and p53-mediated apoptosis induction.

The activation of macropinocytosis by PDAC tumors as an adaptation to nutrient deficiency has been proposed as a potential therapeutic target for the development of novel antitumor modalities against PDAC [27]. In the present study, PDAC-associated PSCs exhibited higher levels of macropinocytosis than did control PSCs, presumably explaining the enhanced virus sensitivity seen in PDAC-associated PSCs. Regarding the mechanism of macropinocytosis activation in PDAC, Kamphorst et al. showed that macropinocytosis is employed to induce the accumulation of essential amino acids, including glutamine, in both PDAC cells and surrounding stromal cells [9]. Lee et al. showed that glutamine deprivation induces macropinocytosis activation in PDAC cells via the epidermal growth factor receptor signaling pathway [28]. Separately, Zhang et al. demonstrated that glutamine depletion induces potentiation of macropinocytosis in PDAC-activated CAFs via the AMP-activated protein kinase signaling pathway [12]. Although the underlying mechanism of macropinocytosis activation in PDAC-associated PSCs remains unclear, metabolic modulation to induce macropinocytosis in PDAC cells and PDAC-associated PSCs may provide a potential strategy to promote the sensitivity to oncolytic adenoviruses of PDAC tumors with stromata.

We observed that PDAC-associated PSCs are more sensitive to p53-armed OBP-702 than to non-armed OBP-301, a difference that appears to reflect the activation of p53-mediated apoptosis. These findings suggest the therapeutic potential of p53 activation against PDAC-associated PSCs. Regarding the role of p53 in CAFs, no genetic alterations in the *p53* gene have been detected in human PDAC-associated PSCs [29]. However, Bar et al. demonstrated that CAFs are more sensitive to p53 depletion (as induced by CM from human cancer cells) than are normal fibroblasts [30]. Arandkar et al. showed that lung cancer-derived CAFs exhibit a decreased phosphorylation of p53 protein compared to normal lung fibroblasts [31]. We also recently reported that gastric cancer-derived CAFs exhibit decreased phosphorylation of p53 protein compared to normal gastric fibroblasts (GFs) [32]. Together, these findings suggest that p53 function is dysregulated in CAFs. In contrast, regarding the therapeutic potential of p53 activation in CAFs, Saison-Ridinger et al. demonstrated that nutlin-3a, an inhibitor of the E3 ubiquitin ligase encoded by *Murine double minute 2*, induces p53 activation and subsequent reprogramming of activated PSCs to a quiescent state, leading to a depletion of the tumor stroma [33]. We recently showed that OBP-702 infection results in stronger attenuation of viability in CAFs than in GFs, a difference reflecting the induction of apoptosis [32]. Thus, PDAC-associated PSCs may possess abnormalities in p53 function, rendering these cells more sensitive to OBP-702-mediated p53 activation and the induction of apoptosis than are non-activated PSCs.

Previous work has suggested that oncolytic adenoviruses might serve as a novel antitumor modality for treating PDAC tumors [34, 35]. However, the desmoplastic stroma in a PDAC tumor is expected to function as a physical barrier against the spread of oncolytic adenovirus, potentially leading to attenuation of the antitumor effect of oncolytic virotherapy [35]. Therefore, depletion of the dense stroma likely will be needed to promote the spread of viruses into PDAC tumors. In the present study, we demonstrated that p53-armed OBP-702 has the therapeutic potential to suppress the proportion of both PDAC cells and PDAC-associated PSCs, as assessed by both 3D culture models and xenograft tumor models. These findings suggest that OBP-702-mediated p53 activation in PDAC tumors is a potent therapeutic strategy to decrease the viability of both PDAC cells and PDAC-associated PSCs; we hypothesize that this decrease in viability will facilitate the spread of OBP-702. Recent evidence has suggested the heterogeneity of PSC-derived CAFs, including myofibroblastic CAFs and inflammatory CAFs, in the TME of PDAC [36]; such heterogeneity presumably contributes to the complicated network employed for crosstalk between PDAC cells and PSC-derived CAFs. Given that the relationship between OBP-702 sensitivity and CAF subtype remains unclear, further experiments are warranted to evaluate the therapeutic potential of OBP-702 against heterogeneous CAF subtypes in PDAC tumors with stromata.

In the context of drug development, it will be important to investigate the safety and feasibility of OBP-702 treatment in preclinical and clinical settings. We recently demonstrated that intraperitoneal administration of OBP-702 was safe in tumor-bearing mice with intraperitoneal metastasis of gastric cancer [37]. Intraperitoneal administration of OBP-702 did not cause severe adverse events as assessed by blood biochemistry, body weight, and tissue damage in vital organs [37]. In contrast, intratumoral administration of OBP-702 suppressed the tumor growth in murine PDAC tumor models without affecting body weight in mice [19, 38, 39]. These findings suggest that intratumoral administration of OBP-702 is safe in tumor-bearing mice. Recently, we demonstrated that intratumoral administration of non-armed OBP-301 was well tolerated by patients with esophageal cancer who are unfit for standard treatment [40]. Therefore, intratumoral administration of OBP-702 would provide a novel treatment strategy for PDAC tumors.

OBP-702 monotherapy did not completely eradicate PDAC tumors with fibrotic stromata (Fig. 6D–F). These findings suggest the potential utility of combination therapy employing OBP-702 and other drugs. Regarding the therapeutic potential of combination therapy with OBP-702 in PDAC tumors, we recently demonstrated that OBP-702 significantly enhances the antitumor effect of immune checkpoint inhibitors (ICIs), including anti-programmed cell death 1 antibody and anti-programmed cell death ligand 1 antibody in murine PDAC tumors [38, 39]. OBP-702 induces p53-mediated immunogenic cell death and suppression of immunosuppressive myeloid-derived suppressor cells. PDAC-associated PSCs also have been shown to play a pivotal role in the tumor progression and immunosuppressive TME [26]. Given that OBP-702 suppresses the activation of PDAC-associated PSCs in PDAC tumors via macropinocytosis-mediated enhancement of virus sensitivity, combination therapy with OBP-702 and ICIs may be a promising antitumor strategy for treating PDAC tumors with fibrotic stromata.

In conclusion, we demonstrated that PDAC-associated PSCs exhibit enhanced macropinocytosis-mediated virus entry compared to control PSCs. Macropinocytosis activation promotes sensitivity to oncolytic adenoviruses in PDAC-activated PSCs, an effect that appears to be mediated via induction of p53-mediated apoptosis. Taken together, our data indicate that PDAC-associated PSCs with enhanced macropinocytosis are a potential therapeutic target for treating PDAC tumors with oncolytic adenoviruses.

## Supplementary information

Supplementary Figure 1-4

## Data Availability

All data generated or analyzed during this study are included in the main text. Further enquiries should be directed to the corresponding author.
