# Supplementary material for: p53-armed oncolytic adenovirus induces apoptosis in pancreatic cancer-associated stellate cells via macropinocytosis
Source: Cancer Gene Ther. 2025 Nov 28;33(2):223–35. doi: 10.1038/s41417-025-00989-3 (PMC12916325; doi:10.1038/s41417-025-00989-3)
Supplement: Supplementary file 1 — Supplementary Figure 1-4 [file 41417_2025_989_MOESM1_ESM.pdf]

Supplementary Information

p53-armed oncolytic adenovirus induces apoptosis in pancreatic cancer-associated stellate cells via macropinocytosis

Takeyoshi Nishiyama[1], Hiroshi Tazawa[1,2], Yasuo Nagai[1], Ryohei Shoji[1], Yoshinori Kajiwara[1], Naoyuki Hashimoto[1], Yosuke Takahashi[1], Satoru Kikuchi[1], Shinji Kuroda[1], Toshiaki Ohara[1,3], Kazuhiro Noma[1], Ryuichi Yoshida[1], Yuzo Umeda[1,4], Hiroyoshi Y. Tanaka[5], Mitsunobu R. Kano[5], Atsushi Masamune[6], Yasuo Urata[7], Shunsuke Kagawa[1], Toshiyoshi Fujiwara[1]

Departments of [1]Gastroenterological Surgery and [3]Pathology and Experimental Medicine, Okayama University Graduate School of Medicine, Dentistry and Pharmaceutical Sciences, Okayama 700-8558, Japan. [2]Center for Innovative Clinical Medicine, Okayama University Hospital, Okayama 700-8558, Japan. [4]Department of HBP and Breast Surgery, Ehime University Graduate School of Medicine, Ehime 791-0295, Japan. [5]Department of Pharmaceutical Biomedicine, Okayama University Graduate School of Interdisciplinary Science and Engineering in Health Systems, Okayama 700-8530, Japan. [6]Division of Gastroenterology, Tohoku University Graduate School of Medicine, Miyagi 980-8575, Japan. [7]Oncolys BioPharma, Inc., Tokyo 105-0001, Japan.

Figure S1

Decreased expression of adenovirus receptors in PSCs incubated with PDAC-CM.

Figure S2

Enhancement of OBP-401-mediated GFP expression in PSCs incubated with tumor cell-CM.

Figure S3

Representative images of immunohistochemical staining for cytokeratin and p53 in the 3D tissues obtained with BxPC-3 cells and hPSC-5 cells.

Figure S4

Representative images of immunohistochemical staining for cytokeratin and Ad5-hexon in BxPC-3 + hPSC-5 tumors treated with mock (PBS), OBP-301, or OBP-702.

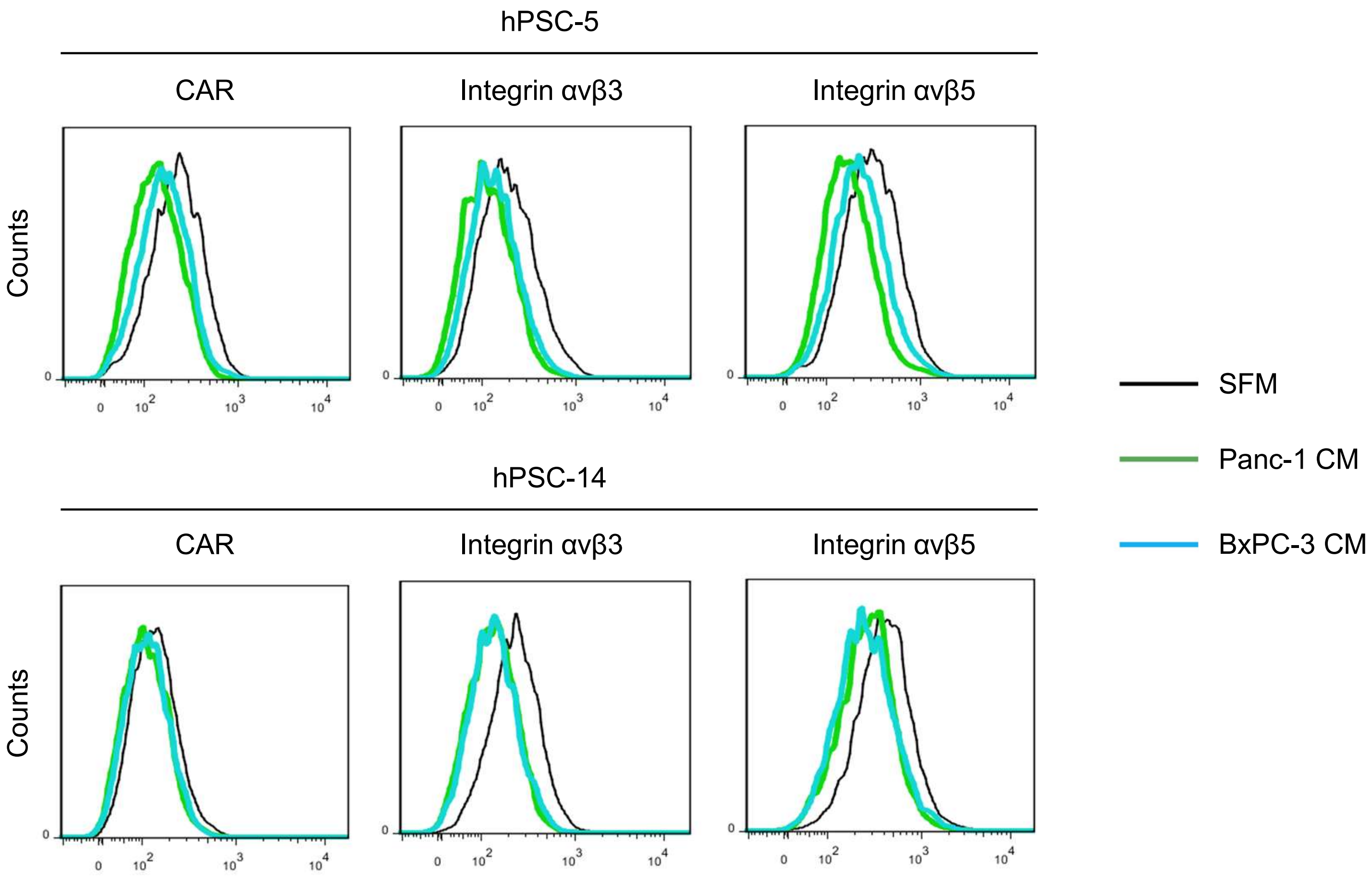

Figure S1. Decreased expression of adenovirus receptors in PSCs incubated with PDAC-CM. Expression of CAR and integrins αvβ3 and αvβ5 on the surface of hPSC-5 and hPSC-14 cells incubated in the presence of SFM or PDAC-CM was assessed by flow cytometry.

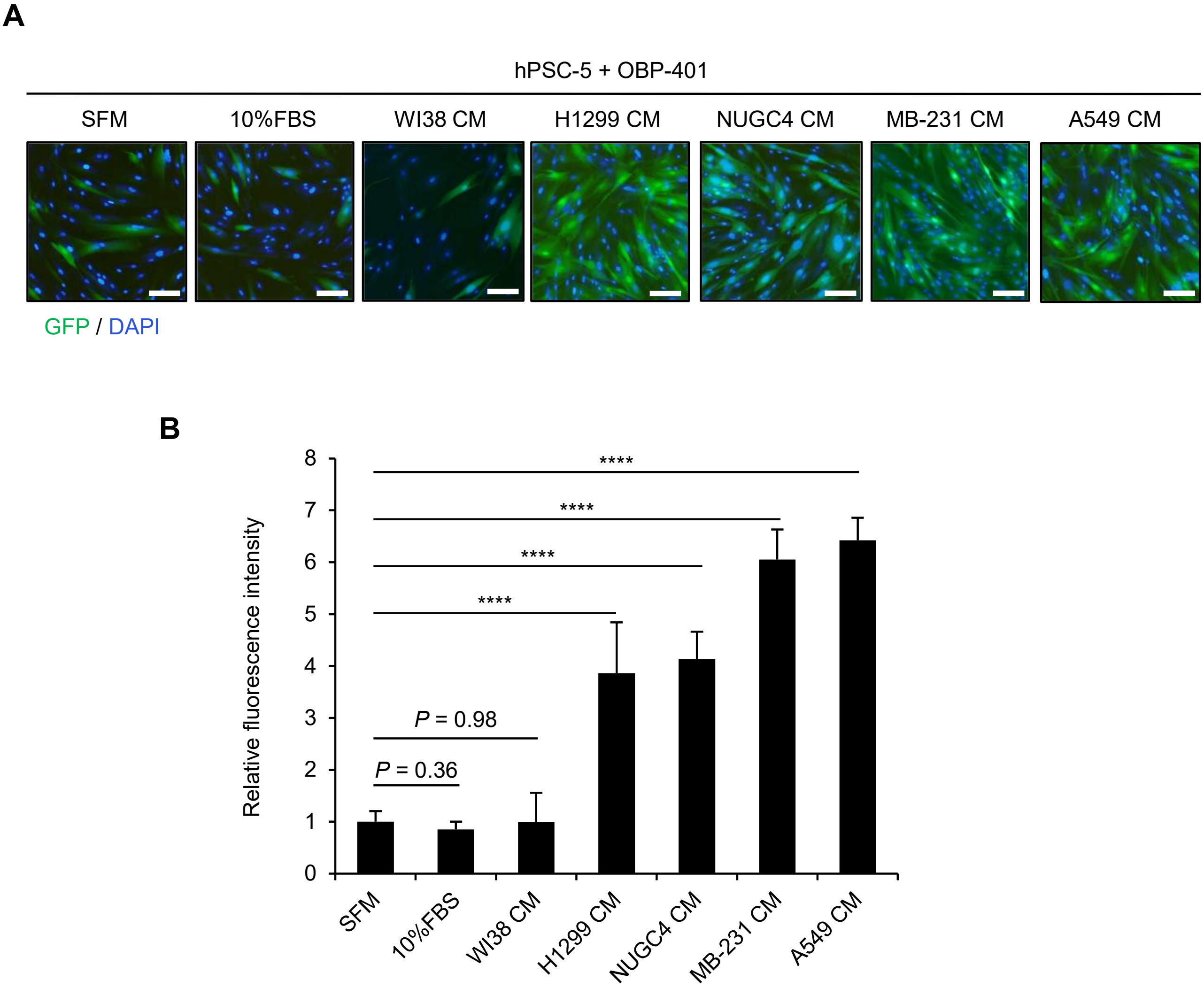

Figure S2. Enhancement of OBP-401-mediated GFP expression in PSCs incubated with tumor cell-CM. A, Representative images of GFP fluorescence in hPSC-5 cells following infection with OBP-401 (MOI 100) for 48 h in the presence of SFM, fresh medium containing 10% FBS, or CM from human fibroblasts (WI38) and human cancer cells (H1299, NUGC4, MB-231, A549). Scale bars: 200 µm. **B**, Relative fluorescence intensity of GFP was analyzed using ImageJ software. Data are expressed as mean ± SD (n = 3). The statistical significance of differences between two groups was determined using the two-tailed, non-paired Student's *t*-test. ****, $P < 0.0001$ (vs SFM).

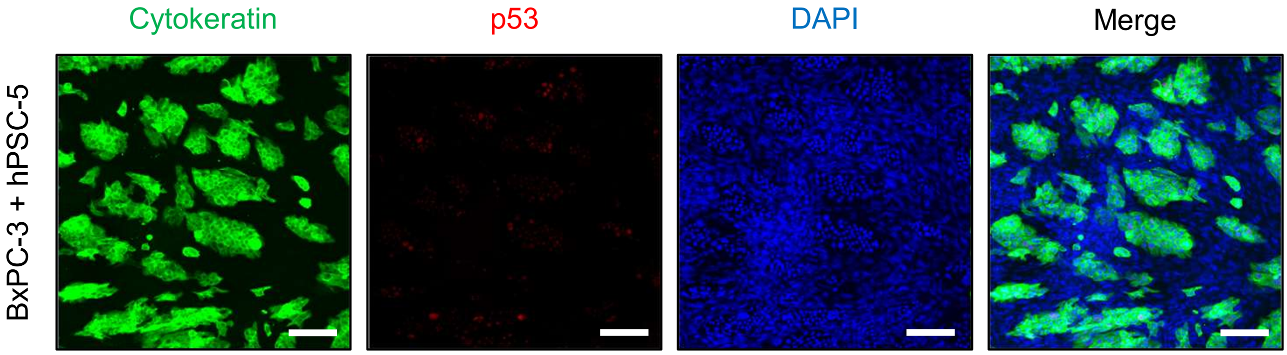

Figure S3. Representative images of immunohistochemical staining for cytokeratin and p53 in the 3D tissues obtained with BxPC-3 and hPSC-5 cells. Scale bars: 200 µm.

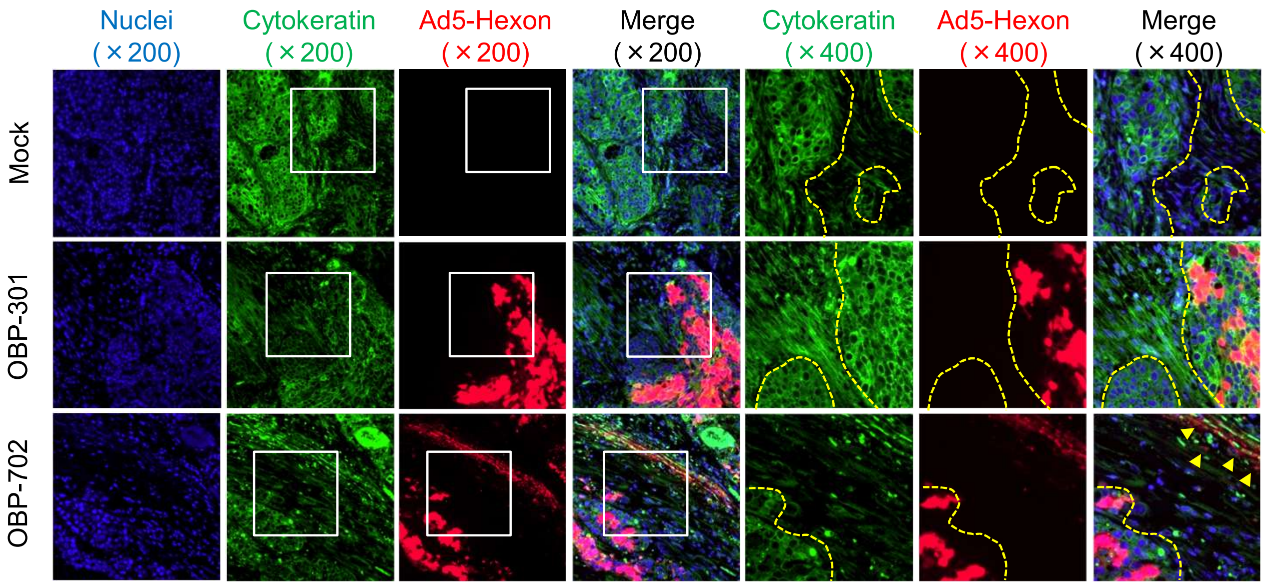

Figure S4. Representative images of immunohistochemical staining for cytokeratin and Ad5-hexon in BxPC-3 + hPSC-5 tumors treated with mock (PBS), OBP-301, or OBP-702. Yellow arrowheads represent Ad5-hexon-positive cells in the tumor stroma.
